# Supplementary material for: Printed origami thermoelectric generator achieves > 20 Wm−² from low-grade heat via material and process design
Source: Nat Commun. 2026 Jan 31;17:1259. doi: 10.1038/s41467-026-68852-z (PMC12864769; doi:10.1038/s41467-026-68852-z)
Supplement: Supplementary file 1 — Supplementary information [file 41467_2026_68852_MOESM1_ESM.pdf]

Supplementary Information

Printed origami thermoelectric generator achieves > 20 Wm^{-2} from low-grade heat via material and process design

Nan Luo[1], Zirui Wang[1], Ajay Kumar Verma[1], Muhammad Irfan Khan[1], Leonard Franke[1,2], Jiayi Liu[1], Alexei Nefedov[3], Marc Schneider[4], Holger Geßwein[5], Erich Müller[6], Kirsten Drüppel[7], Tobias Weingärtner[8], Yolita M. Eggeler[6], Uli Lemmer[1,4,] and Md Mofasser Mallick[1,2,*]*

[1]Light Technology Institute, Karlsruhe Institute of Technology (KIT), 76131 Karlsruhe, Germany.

[2]Varmo UG (haftungsbeschränkt), Hermann-von-Helmholtz-Platz 6, 76344 Eggenstein-Leopoldshafen, Germany

[3]Institute of Functional Interfaces, Karlsruhe Institute of Technology (KIT), 76344 Eggenstein-Leopoldshafen, Germany

[4]Institute of Microstructure Technology, Karlsruhe Institute of Technology (KIT), 76344 Eggenstein-Leopoldshafen, Germany

[5]Institut für Angewandte Materialien (IAM-ESS), Karlsruhe Institute of Technology (KIT), Hermann-von-Helmholtz-Platz 1D, 76344 Eggenstein-Leopoldshafen, Germany

[6]Laboratory for Electron Microscopy, Karlsruhe Institute of Technology (KIT), 76131 Karlsruhe, Germany

[7]Institute for Applied Geosciences, Karlsruhe Institute of Technology (KIT), 76131 Karlsruhe

[8]Institut für Angewandte Materialien (IAM-AWP), Karlsruhe Institute of Technology (KIT), Hermann-von-Helmholtz-Platz 1D, 76344 Eggenstein-Leopoldshafen, Germany

*Corresponding authors

Email: uli.lemmer@kit.edu; mofasser.mallick@kit.edu

Note S1: Rietveld refinement of XRD patterns of the $Ag_2(Se_{1-x}S_x)_{1.05}$ films

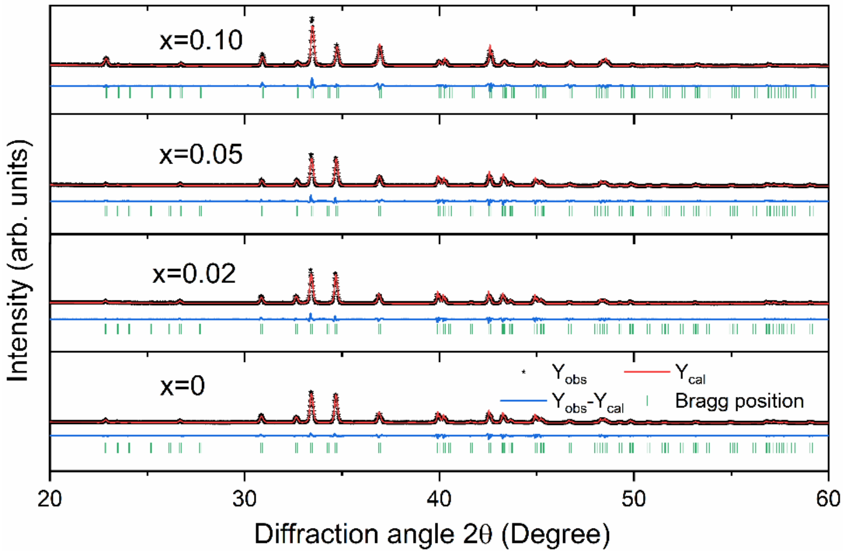

Fig. S1: XRD patterns of the printed films.

Rietveld refinement of room temperature XRD patterns for the $Ag_2(Se_{1-x}S_x)_{1.05}$ films

Note S2: Elemental mapping of the printed $Ag_2(Se_{1-x}S_x)_{1.05}$ films

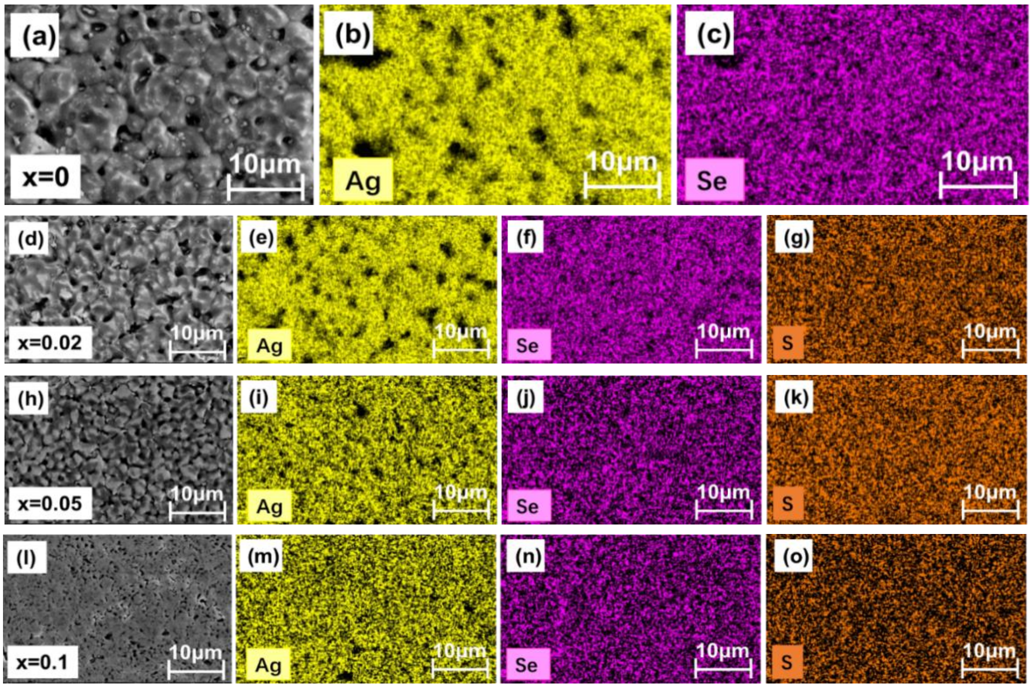

Fig. S2.Microstructure and chemical distribution of $Ag_2(Se_{1-x}S_x)_{1.05}$ printed films.

EDXS maps of the elements Ag, Se and S of $Ag_2(Se_{1-x}S_x)_{1.05}$ films for x=0 (a)-(c);x=0.02(d)-(g);x=0.05(h)-(k);x=0.1(l)-(o).

Quantitative analysis of the n-type printed film's chemical composition through EDXS maps indicates that Ag constitutes 64.4 at. %, Se constitutes 35 at.%, and S comprises 0.69 at.%. While we can observe the existence of S, it is difficult to measure the exact composition due to the low doping concentration.

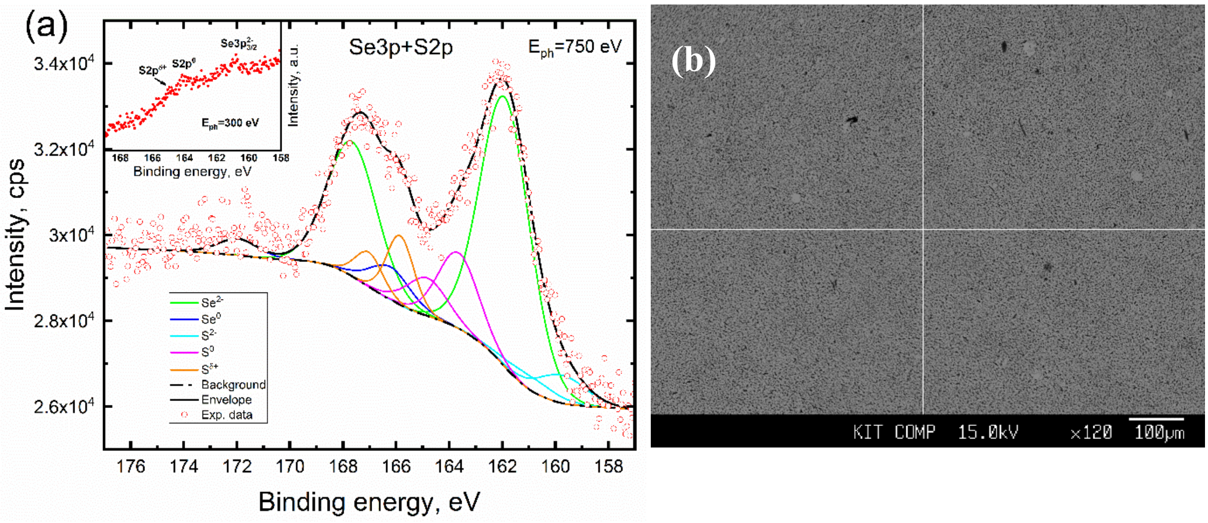

Fig. S3: XPS data of the printed film.

(a) *Se3p/S2p* XP spectrum of printed $Ag_2(Se_{1-x}S_x)_{1.05}$ film measured at excitation photon energy of 750 eV: red circles - experimental spectrum, thick black line – envelope spectra. The thin lines in b correspond to individual doublets of different *Se3p/S2p*- components. Inset: The same XP spectrum measured at excitation photon energy of 300 eV. (b) EPMA backscattered electron (BSE) image of the printed $Ag_2(Se_{1-x}S_x)_{1.05}$ film.

Table S1: Elemental composition of the printed $Ag_2(Se_{0.98}S_{0.02})_{1.05}$ film at four different points using EPMA

**Printed $Ag_2(Se_{0.98}S_{0.02})_{1.05}$**	**Point 1**	**Point 2**	**Point 3**	**Point 4**
	wt.%			
Se	23.17	22.52	25.13	27.21
S	0.21	0.22	0.17	0.20
Ag	68.30	63.41	70.47	70.31
Total	91.67	86.15	95.76	97.71
	at. %			
Se	31.45	32.42	32.58	34.37
S	0.69	0.76	0.53	0.62
Ag	67.86	66.82	66.89	65.01
Total	100.00	100.00	100.00	100.00
NF	0.03	0.03	0.03	0.03

Note S3: Mechanical flexibility of the printed n-type leg

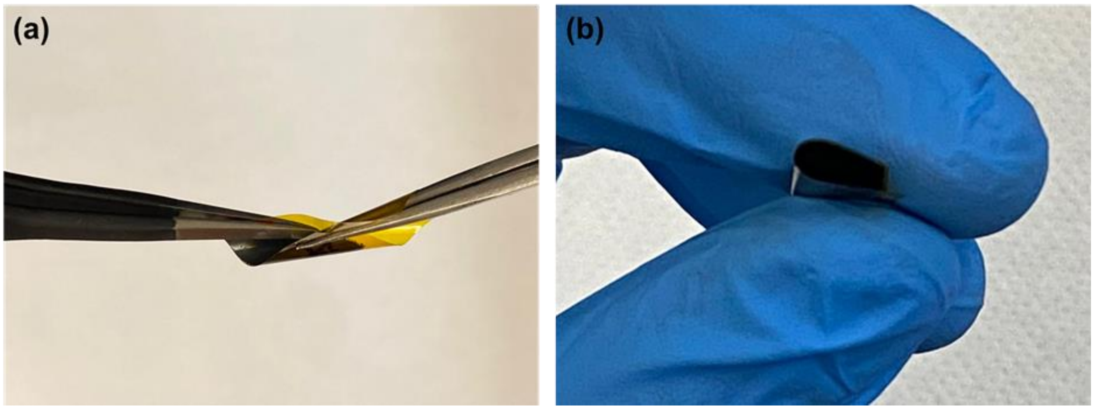

Fig. S4: Mechanical flexibility of the printed film.

(a) Twisting test; (b) Complete folding of the printed n-type film for x=0.02, demonstrating its high flexibility.

The printed n-type $Ag_2(Se_{1-x}S_x)_{1.05}$ demonstrates excellent mechanical flexibility, maintaining stable resistance after twisting and complete folding.

Note S4: Thermoelectric properties of n-type leg with varying sulfur content

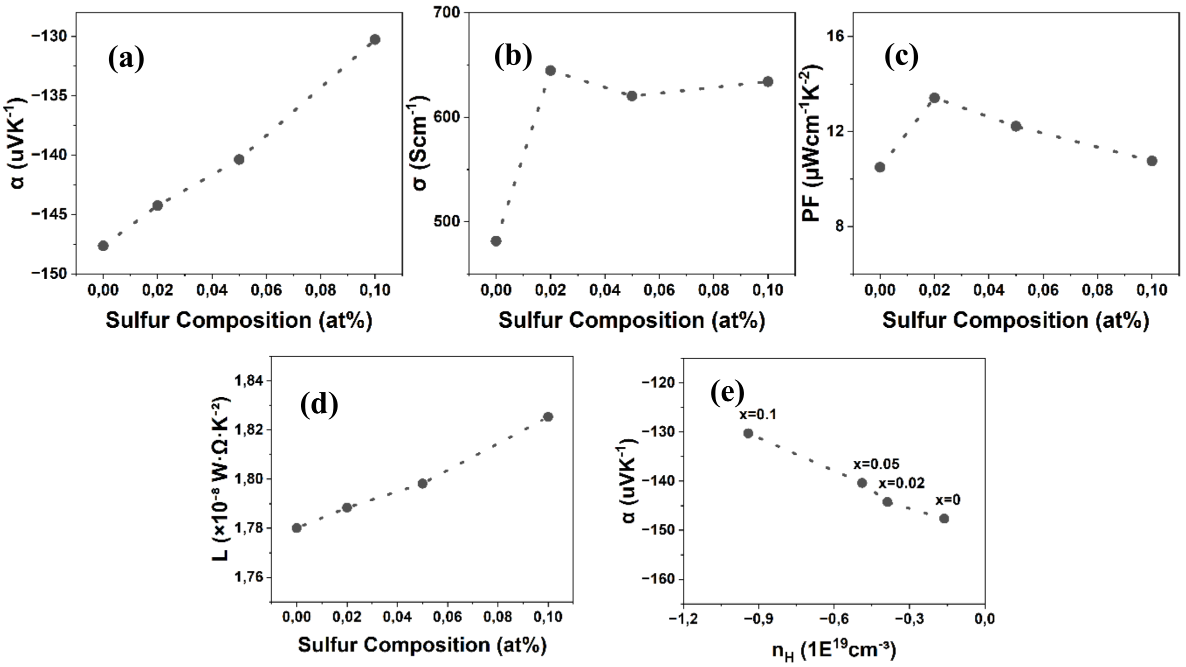

Fig. S5: TE properties of n-type material at 303K with 'x'..

(a) *Seebeck coefficient α* ***(b)*** *Electrical conductivity* σ *and* ***(c)*** *Power factor* $\alpha^2\sigma$*. (d) Lorenz number of n-type material with varying sulfur content (e) Relationship between Seebeck coefficient α and charge carrier concentration* n_H.

Fig. S5 illustrates the thermoelectric properties of the n-type leg - $Ag_2(Se_{1-x}S_x)_{1.05}$ as a function of sulfur content (at. %) at 303 K. Incorporating sulfur significantly increases the electrical conductivity (σ), which reaches a maximum value of 644.85 S·cm^{-1} at x = 0.02. Meanwhile, the Seebeck coefficient (α) decreases with increasing sulfur content. However, the calculated power factor ($\alpha^2\sigma$) reaches its maximum 13.42 μWcm^{-1}K^2 also at x = 0.02, indicating an optimal balance between conductivity and Seebeck coefficient at this composition. Thermal conductivity was estimated for the x = 0.02 composition based on the measured value of a non-pressed film, in order to predict its ZT at room temperature using the following.

$$\kappa_{eff} = \kappa_{bulk}(1 - P)$$

Where κ_{eff} and κ_{bulk} are the thermal conductivity of the printed film and bulk Ag_2Se. P is the porosity. The porosity of the non-pressed and pressed film for x=0.02 estimated to be $P_{60} = 60\ \%$ and $P_{45} = 45\ \%$ respectively. The estimated thermal conductivity of the pressed printed film $\kappa_{eff,49}$ for x=0.02 can be calculated using the following expression;

$$\kappa_{eff,45} = \kappa_{eff,60}\frac{(1 - P_{45})}{(1 - P_{60})}$$

Thermal conductivity of the non-pressed film $\kappa_{eff,60}$ (0.43 Wm^{-1}K^{-1}) is measured by thin film analyser. Hence, $\kappa_{eff,45} = 0.59$ Wm^{-1}K^{-1} at room temperature and corresponding ZT=0.7.

Note S5: Reproducibility and stability of printed $Ag_2(Se_{0.98}S_{0.02})_{1.05}$ film

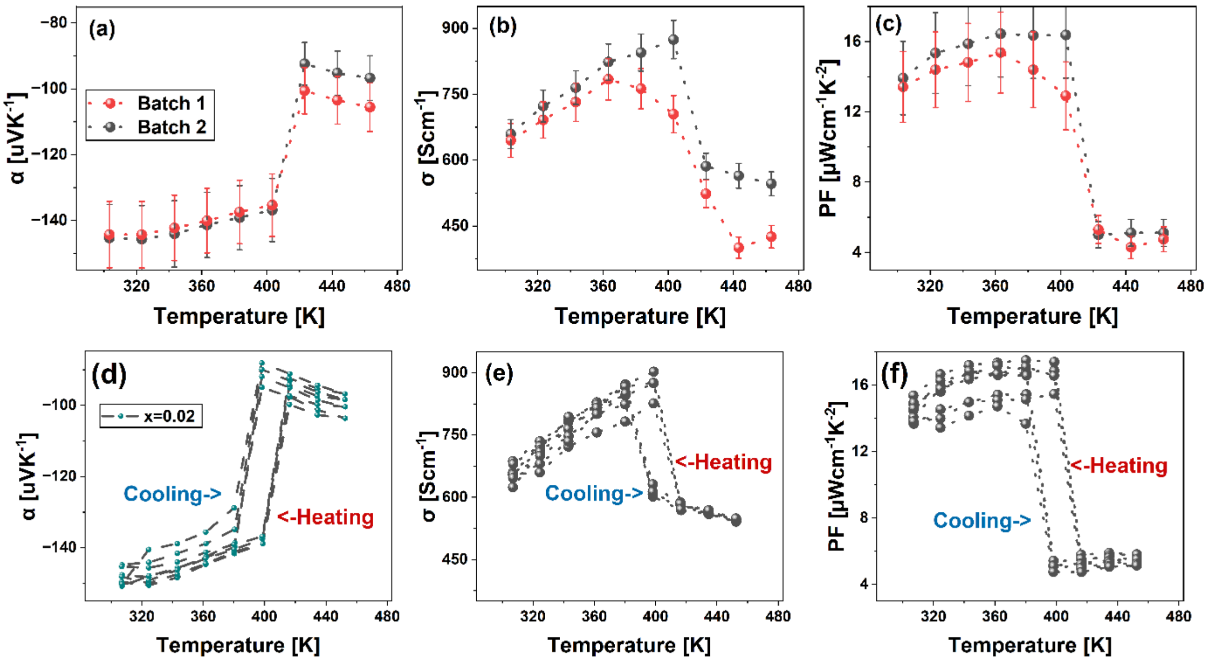

Fig. S6: TE properties of the printed film for x=0.02.

Comparison of TE properties of n-type printed films for 'x=0.02' prepared from two different batches of inks (I and II): (a) Seebeck coefficient α (b) Electrical conductivity σ *and (c) Power factor* $\alpha^2\sigma$*. (d-f) TE properties of the printed* $Ag_2(Se_{0.98}S_{0.02})_{1.05}$ *film during 5 heating/cooling cycles.*

Two printed $Ag_2(Se_{0.98}S_{0.02})_{1.05}$ films were prepared from two different batches of inks. The TE performance of films are calculated and compared in Fig. S6 (a-c). It is found the overall power factor value remain within 10 %. The TE performance of the printed $Ag_2(Se_{0.98}S_{0.02})_{1.05}$ film was also measured in 5 heating/cooling cycles (c.f. Fig. S6 (d-f)). There is small hysteresis found between heating and cooling. However, the results show the film thermally stable and produce similar performance.

Note S6: Thermoelectric properties of printed $Ag_2(Se_{0.99}S_{0.01})_{1.05}$ film

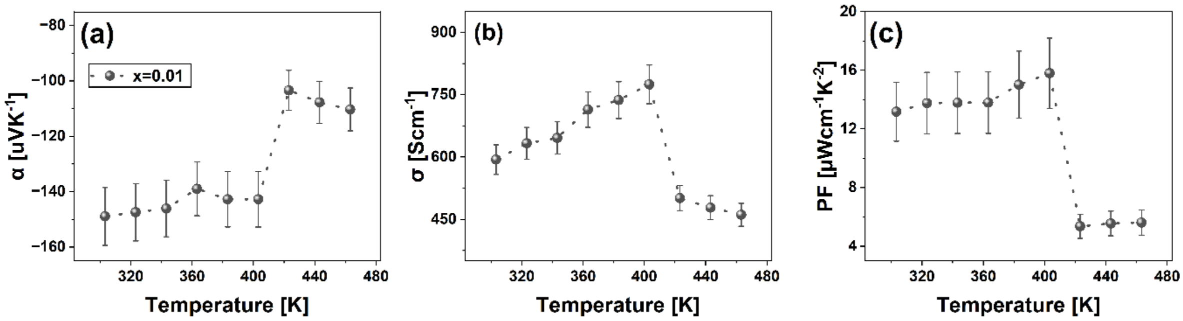

Fig. S7: TE properties of n-type printed films for 'x=0.01' prepared film.

(a) Seebeck coefficient α (b) Electrical conductivity σ *and (c) Power factor* $\alpha^2\sigma$*.*

Note S7: Thermoelectric properties of printed Sb-Bi-Te p-type film

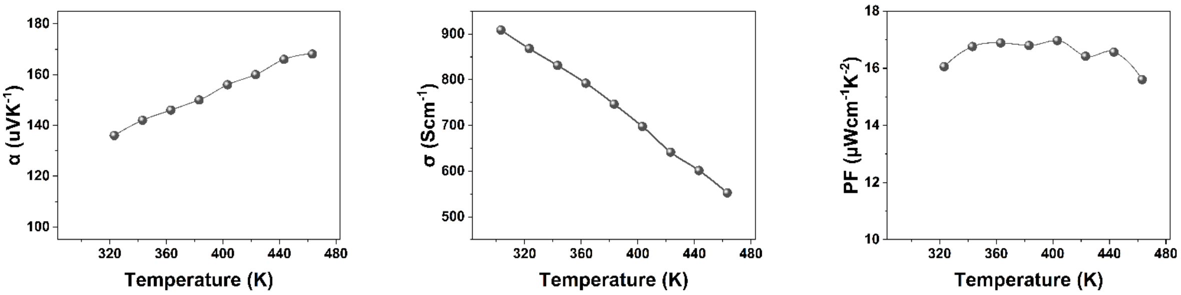

Fig. S8: Temperature-dependent TE properties of p-type material.

(a) Seebeck coefficient α (b) Electrical conductivity σ *(c) Power factor* $\alpha^2\sigma$.

Fig. S8 presents the TE properties of p-type material - $Bi_{0.5}Sb_{1.5}Te_3$ after sintering and hot-pressing. A power factor of 16 $\mu Wm^{-1}K^{-2}$ achieves at 320 K.

Note S8: Structures of origami-TEGs

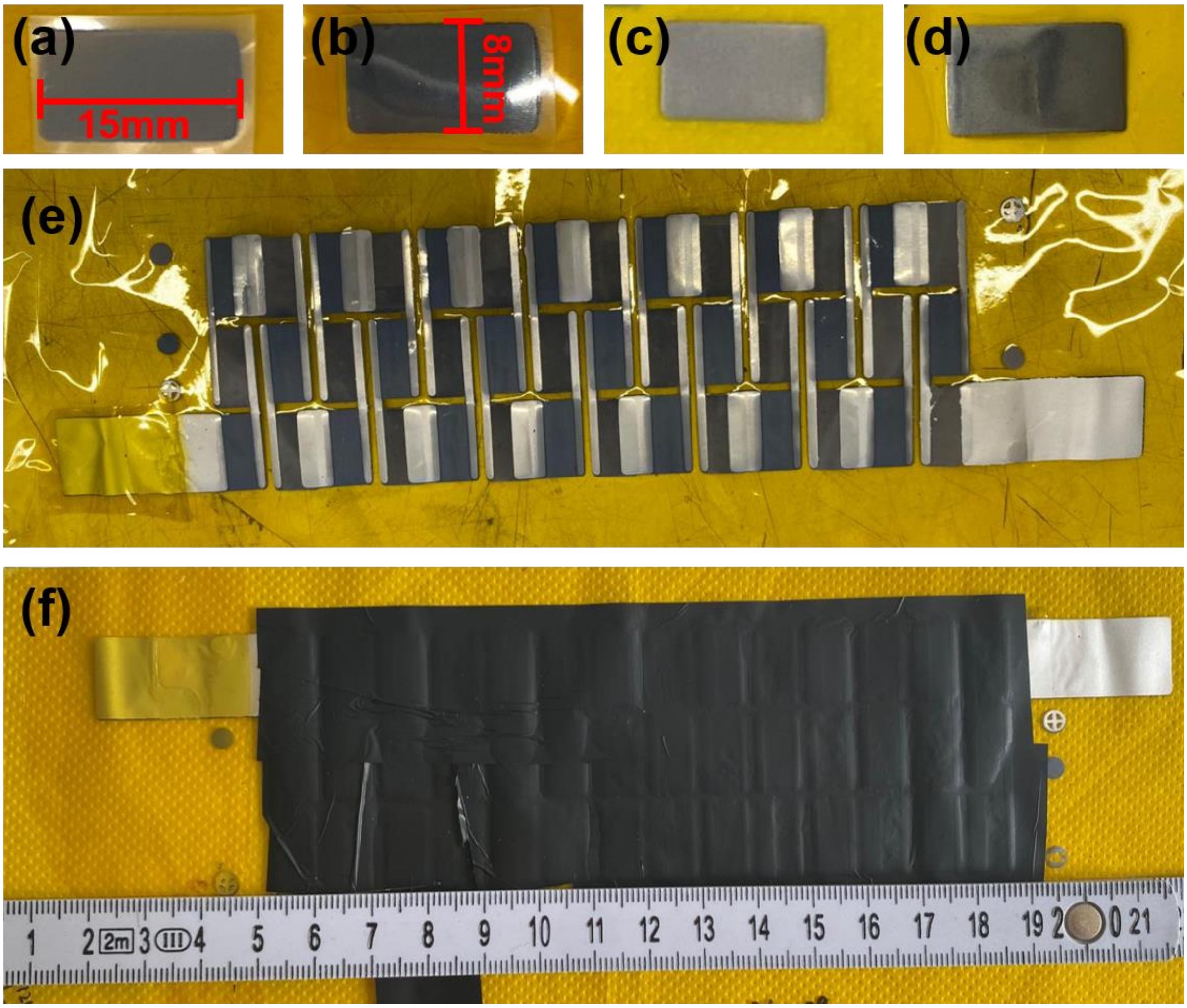

Fig. S9:Images of the printed TE legs and origami TEG.

(a) non-hot-pressed n-type leg; (b) hot-pressed n-type leg; (c) non-hot-pressed p-type leg; (d) hot-pressed p-type leg; (e)hot-pressed origami-TEG; (f) Carbon encapsuled origami -TEG

Fig. S9 (a)-(d) compare the non-hot-pressed and hot-pressed n-type and p-type legs, demonstrating that hot pressing creates a smooth and flat surface. Fig. S9 (e) shows the origami-TEG, revealing that the printed TEG consists of 21 thermocouples. Each TE leg has dimensions of 8 mm × 15 mm. Fig. S9 (d) shows the encapsulated printed TEG. A 5 µm carbon layer was applied for encapsulation to prevent electrical short circuits during measurement and enhance mechanical stability.

Note S9: COMSOL simulated model of device

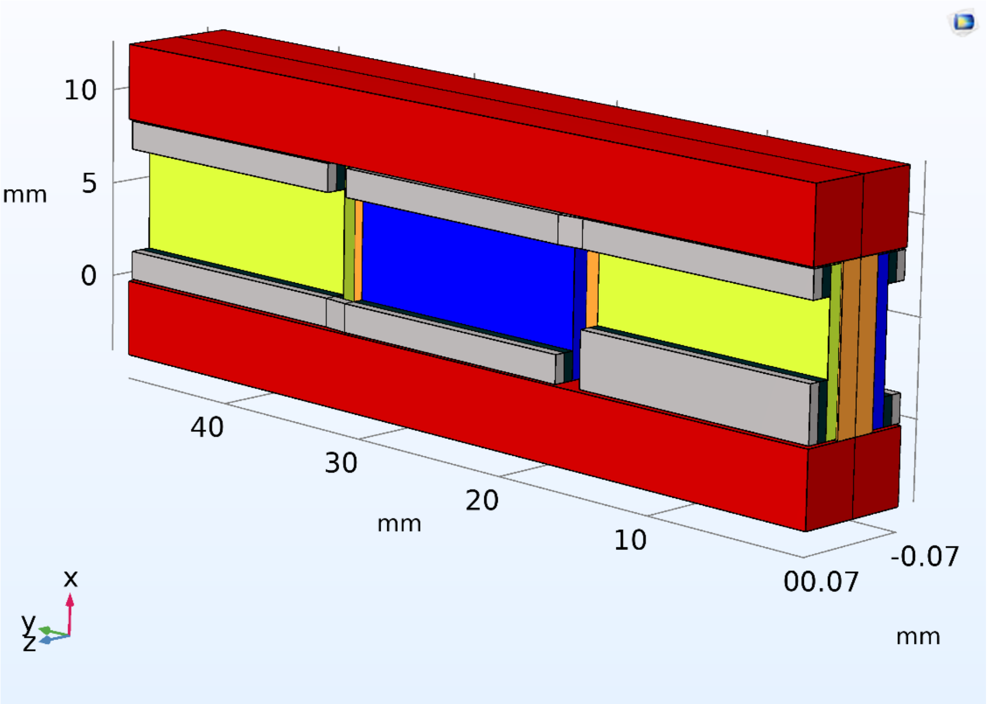

Fig. S10: *Simulated device model*

Yellow part is p-type leg - $Bi_{0.5}Sb_{1.5}Te_3$, blue part is n-type leg - $Ag_2(Se_{0.98}S_{0.02})_{1.05}$, black part is the carbon diffusion barrier, gray part is Ag conductor.

Table S2. Structural parameters of the Origami TEG device used in COMSOL

Table 1 | Structural parameters of the Origami TEG (COMSOL model).

No. of Layers	Material	Thickness (µm)
Layer 1	Kapton (Substrate)	24
Layer 2	p-type ($Bi_{0.5}Sb_{1.5}Te_3$)	17
Layer 3	n-type (Ag_2Se)	14
Layer 4	Diffusion barrier (Carbon ink)	12
Layer 5	Conductor (Ag ink)	10
**Origami TEG**	**-**	**910.67**
